# The Entomopathogenic Fungus *Beauveria bassiana* Shows Its Toxic Side within Insects: Expression of Genes Encoding Secondary Metabolites during Pathogenesis

**DOI:** 10.3390/jof8050488

**Published:** 2022-05-07

**Authors:** Nicolás Pedrini

**Affiliations:** Instituto de Investigaciones Bioquímicas de La Plata (INIBIOLP), CCT La Plata Consejo Nacional de Investigaciones Científicas y Técnicas (CONICET), Universidad Nacional de La Plata (UNLP), Calles 60 y 120, La Plata 1900, Argentina; npedrini@med.unlp.edu.ar

**Keywords:** entomopathogenic fungi, virulence, nonribosomal peptides (NRPS), polyketides (PKS)

## Abstract

Entomopathogenic fungi are extensively used for the control of insect pests worldwide. Among them, *Beauveria bassiana* (Ascomycota: Hypocreales) produce a plethora of toxic secondary metabolites that either facilitate fungal invasion or act as immunosuppressive compounds. These toxins have different chemical natures, such as nonribosomal peptides and polyketides. Even though their precise role is poorly understood, they are usually linked to virulence. These fungal secondary metabolites are produced by the expression of gene clusters encoding the various proteins needed for their biosynthesis. Each cluster includes synthetases for nonribosomal peptides (NRPS), polyketides (PKS), or hybrid NRPS–PKS genes. The aim of this review is to summarize the information available from transcriptomics and quantitative PCR studies related to the expression of *B. bassiana* NRPS and PKS genes inside different insects as the infection progresses; as for the host immune response, to help understand the mechanisms that these toxins trigger as virulence factors, antimicrobials, or immunosuppressives within the context of a fungus–insect interaction.

## 1. Introduction

**Figure 1:**
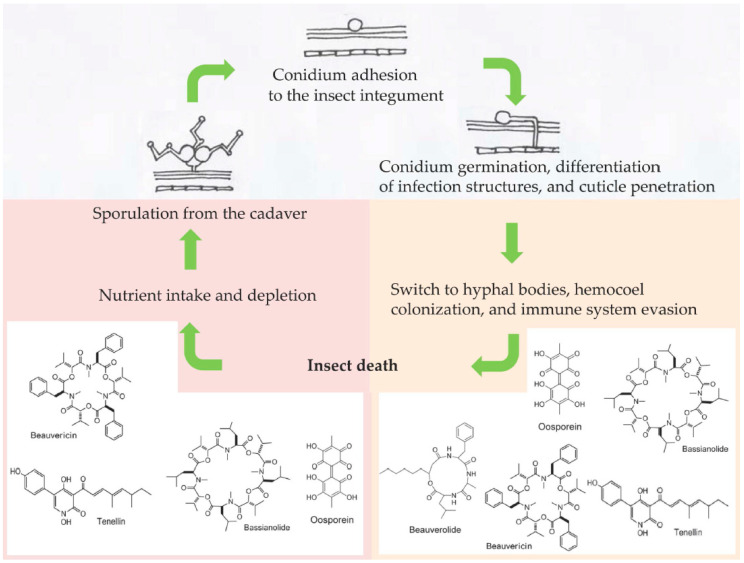
Schematic showing some of the secondary metabolites produced by *Beauveria bassiana* during the infection process in an insect host. Examples of compounds produced before insect death (potentially involved in virulence) and after insect death (potentially acting as antimicrobials) are shown.

The fungus *Beauveria bassiana* (Ascomycota: Hypocreales) is a generalist (broad-spectrum host range) insect pathogen able to infect nearly 1000 insect species [1]. As for other entomopathogenic fungi of the order Hypocreales, the main route of infection for *B. bassiana* is the penetration of the insect cuticle, which represents the first encounter and barrier between the fungus and host [2]. Upon the adhesion to and recognition of the insect surface, *B. bassiana* deploys a combination of biochemical and mechanical tools to make its way through the insect integument into the hemocoel [3] (Figure 1). Once the fungus reaches this nutrient-rich environment, the mycelium switches to a specialized yeast-like cell phenotype; in invertebrate pathology, they are often referred as hyphal bodies or blastospores when they are produced artificially in culture media. At this stage, the insect host has very little chance of surviving the fungal infection despite the activation of the immune response (humoral and cellular) as a last-ditch attempt to overcome the fungus [4]. A successful fungal infection will then depend on the concerted combination of several events, one of the main ones being the production of a plethora of toxic secondary metabolites [5] that can either facilitate the fungal invasion [6] or act as immunosuppressive compounds, fighting against host defenses [7]. These secondary metabolites can have many different chemical natures, and include nonribosomal peptides and polyketides. *Metarhizium* spp. mainly produce destruxins (cyclic hexadepsipeptides), and *Beauveria* spp. synthesize beauvericin and bassianolide (cyclooligomeric nonribosomal peptides), a variety of beauverolides (cyclic peptides), oosporein (dibenzoquinone), bassiatin (diketomorpholine), and tenellin (2-pyridone) [5] (Figure 1). Even though the precise role of secondary metabolites is poorly understood, they are usually linked to the virulence of fungal strains [2,4,5,7]. The aim of this review is to summarize the information available from transcriptomics (RNA-seq) and quantitative PCR (qPCR) studies related to the expression of *B. bassiana* genes involved in toxin production inside different insect orders as the infection progresses, to help understand how these toxins participate as virulence factors, antimicrobials, or immunosuppressives within the context of a fungus–insect interaction.

## 2. Role of Secondary Metabolites as Virulence Factors: What We Know and What We Ignore

Searching the information available reveals some clues, but no conclusive evidence, that entomopathogenic fungal secondary metabolites are produced within the insect as part of the infection process. A metabolomics approach found differences in secondary metabolite production on both live and dead tissues, assigning different purposes to different compounds; i.e., beauverolides are involved in killing the host, and destruxins mainly function as antimicrobials [8]. Oosporein also acts as an antimicrobial compound [9], but also promotes infection, probably by reducing the number of insect hemocytes, with the consequent alteration of the humoral immune system [10]. Entomopathogenic fungal secondary metabolites have been isolated from mycelia cakes, free-cell cultures [11,12], and also pooled insects infected with either *Metarhizium* spp. [13,14] or *Beauveria* spp. [15]. However, the analysis of individual mycosed insects rather than pooled samples is essential to provide an indication of the expression pattern of secondary metabolites during the time course of fungal infection [16]. A major hurdle to this is that the sensitivity of current analytical techniques does not permit the detection of the few fungal molecules that are expected to be produced inside the insect hemocoel. In this regard, analyzing the expression of genes involved in their biosynthesis when the fungus grows within its insect host could help to better elucidate their roles in pathogenesis.

Secondary metabolites are synthesized from gene clusters, including nonribosomal peptide synthetases (NRPSs), polyketide synthetases (PKSs), and hybrid NRPS–PKS genes [17,18]. Their induction is achieved when entomopathogenic fungi are confronted with whole insects or insect tissues, but several of the known clustered genes have no secondary metabolite assigned, and vice versa. In this regard, over 80% of the putative secondary-metabolite-associated genes identified in *Metarhizium* ssp. and *B. bassiana* have no identified specific products, and their sequences are unique to entomopathogenic fungi [19]. Although bassiacridin and beauverolides are secondary metabolites of currently unknown origin, there are many characterized metabolites with well-known biosynthetic pathways in *B. bassiana*, e.g., tenellin, beauvericin, oosporein, and bassianolide. Functional studies targeting *B. bassiana* NRPS and PKS have assigned very important functions in fungal development and virulence against insect hosts. PKSs are linked to asexual development and cell wall integrity; for example, the *Bbpks11* gene acts in responses to oxidation, high temperature, and UV irradiation [20], and *Bbpks15* is necessary for the formation of conidia and blastospores [21]. Regarding their roles in virulence, the bassianolide synthetase gene (*BbbslS*) is very important [22], the beauvericin synthetase gene (*BbbeaS*) participates, but its role is not key [23], the tenellin synthetase gene (*BbtenS*) does not contribute to virulence [24], and oosporein polyketide synthase (*BbopS1*) seems to directly participate in the evasion of insect immunity, and facilitates fungal growth [25]. More detailed information is available for oosporein expression; the zinc finger protein encoded by *BbSmr1* negatively regulates oosporin production [26] by binding to the promoter region of the *BbbrlA* gene [27]. Using label-free quantitative proteomics, an increased level of oosporein biosynthetic enzymes was reported after the addition of exogenous oosporein into *B. caledonica* cultures [10]. The direct injection of oosporein did not kill the insects, but increased their susceptibility to subsequent fungal infection [10]; similar effects were described for beauverolides and destruxins [16]. For other secondary metabolites, individual applications did not cause significant mortality or macroscopical alterations in insects; however, this does not mean that they are not important, as they may act concertedly, and their exact roles are yet to be understood [16]. The key to unlocking the potential of the secondary metabolites is directly related to understanding and manipulating the complex regulatory networks controlling gene expression in fungi [16]. The biosynthesis of secondary metabolites is an energy-intensive process, and it occurs only under specific ecological conditions, e.g., when an insect’s immune system is attacking the pathogen [28]. Little is known about the environmental or host signals that are responsible for the induction of secondary-metabolite-synthesizing genes over the course of host infection, and there is still a lack of conclusive knowledge about their ecological function. For *B. bassiana*, it was proposed that the environmental signal for NRPS expression mainly comes from the insect host, and thus, there is a group of fast-evolving NRPSs (classified as group II, see below) that are closely associated with pathogenesis [29]. In summary, the scarcity of information about the biosynthetic pathways and products at the molecular level might be the main reason there is no extensive use of secondary metabolite genes from entomopathogenic fungi as a tool for controlling recombinant strains [30,31], although group II NRPS would be the best candidates to test in this regard.

## 3. Expression of Nonribosomal Peptide Synthetases (NRPSs) and Polyketide Synthetases (PKSs) during Pathogenesis

The *B. bassiana* genome harbors 21 NRPS or NRPS-like genes [32]. A comparative genomics study reported a high level of genetic diversity related to virulence between fungal isolates, and some of them show unique NRPS and PKS gene clusters [33]. Phylogenetics combined with comparative genomics also offers a tool useful for predicting the potential roles of *B. bassiana*’s secondary metabolites in effecting the fungus’ fitness and virulence. Of the 21 predicted NRPSs, two of them seem to have unknown functions, and the rest have been putatively categorized into two functional groups: seven NRPSs might be important during both saprophytic and pathogenic lifestyles and, thus, be related to basic metabolism (group I), and 12 NRPSs are likely to be involved in pathogenicity (group II), which includes the well-characterized genes *BbbslS* and *BbbeaS* [29]. In a dual transcriptomics approach between *B. bassiana* ARSEF 2860 and the diamondback moth *Plutella xylostella*, these group II NRPS genes were not found to be expressed [34]. However, the same interacting system (same fungal isolate and host) was assayed by qPCR [29], and all 12 group II NRPS genes were detected in three conditions: fungal growth at 6 or 9 days on artificial complete media, and 6 days after the infection of the diamondback moth. Based on the transcript levels during the last condition, half of the group II NRPSs were likely to be involved in infection, including *BbbeaS*, *BbbslS*, and *BbtenS*, as well as a gene that is speculated to encode a beauverolide synthetase (BBA_08222) [29] (Table 1). Another group II NRPS (BBA_07611) has been identified as highly expressed in the hemocoel of the diamondback moth [29] and by transcriptomics in larvae of the wax moth *Galleria mellonella* [35]. On the other hand, the expression levels of PKAs are much less studied; the oosporein polyketide synthase *BbopS1* is mainly expressed in insect cadavers at 24–48 h after death, reinforcing oosporein’s proposed role as an antimicrobial [26].

The first detailed study on the expression of fungal genes involved in the biosynthetic pathways for secondary metabolites inside insects, as well as the host response, was developed by Lobo et al. [36]. This absolute quantification using dual qPCR allowed them to observe that *B. bassiana*’s expression of toxin genes peaked during the first days of infection, with the toxins perhaps functioning as virulence factors, and later in moribund insects and/or cadavers to protect them from competitive microorganisms [36]. The type of NRPS genes expressed by *B. bassiana* is also related to the fungal isolate: with the same insect as the host (the kissing bug *Triatoma infestans*), the strain GHA mainly expresses *BbbeaS* [36], but the strain Bb-C001 mainly expresses *BbbslS*, with a lower levels of *BbbeaS* and *BbtenS* [37] (Table 1). Both studies were performed at different time points after insect treatment with various concentrations of propagules, either by immersing the insects in conidial suspensions or by injecting them with blastospores, and the toxin profile was the same within each isolate. Despite this difference in toxic signature, the infections with both strains developed with similar virulence. This early evidence allows speculation that different fungal isolates can express different secondary metabolites during insect infection, perhaps in an overlapping manner, but their effects are the same in the general scheme of the infection process, i.e., culminating in host death. However, more case studies are needed to confirm these observations.

## 4. Dimorphic Transition, Fungal Secondary Metabolites, and the Insect Immune System: A Crosstalk Waiting for Elucidation

The morphologic conversion of the dimorphic fungi from hyphae to yeast is required for virulence in fungal pathogens of humans, plants, and insects [38]. In human pathogenic fungi, temperature is the major stimulus for dimorphic transition (from 22–25 °C in the soil to 37 °C in the host), although other factors such as CO_2_, cysteine, and estradiol can influence both conversion and growth at 37 °C [39]. In plant pathogenic fungi, nitrogen sources, some branched-chain amino acids, and the enzyme activity of lipoxygenases and cyclooxygenases produce molecules acting in quorum sensing that contribute to yeast–mycelial dimorphism [40,41]. In insect pathogenic fungi, the stimuli that provoke transitions between penetrating germ tubes to hyphal bodies at the beginning of the infection stage, and from hyphal bodies to mycelia in late infection, are both poorly understood. However, there is evidence that the high osmotic pressure found in the hemocoel may trigger the first switch [4], and a quorum sensing system is involved in the latter [42].

In *B. bassiana*, the dimorphic switch is controlled by multiple signaling systems, including activator genes of central developmental pathways, such as *BbbrlA* and *BbabaA* [27]. Deletion of these genes abolished both submerged blastospore formation in vitro and in vivo, and conidiation in vitro, but did not modify hyphal growth [43]. The entomopathogenic fungal transition from filamentous forms to blastospores is enhanced when conducted in vitro, since liquid fermentations under conditions of elevated glucose and dissolved oxygen are key factors in blastospore development [44]. A comparative genome-wide transcriptomics approach between the filamentous and blastospore growth phases in vitro in *M. anisopliae* found that some genes involved in the biosynthesis of secondary metabolites were upregulated in blastospores [45]. The null mutant of *BbOpS1* (encoding a PKS from oosporein biosynthetic gene cluster in *B. bassiana*) resulted a less virulent strain and impaired blastospore production in vivo [25]. However, there is no information about the NRPS of *B. bassiana* that were functionally characterized by reverse genetics [22,23,24], regarding their role in dimorphic transitions. Thus, this should be the next step towards linking this process with virulence. Although there is abundant information about secondary metabolites and other small molecules in dimorphic transitions and virulence in human pathogenic fungi [46,47,48], it is still an underexplored topic in entomopathogenic fungi. A detailed study by Boucias et al. [42] detected an in vivo quorum sensing system involved in *M. rileyi* transition from yeast-like cells to mycelia, a process that begins with the host tissue invasion stage and then culminates with insect death. These authors demonstrated that neither the insect molting hormone, which prevents metamorphosis, nor some well-characterized quorum sensing modulators of these transitions in human pathogenic fungi, are involved in such a transition [42]. Thus, this quorum sensing activity seems to be unique of this particular species of entomopathogenic fungus, and is unrelated to any known fungal quorum sensing system. It is clear that the transition from hyphal bodies to mycelia is a multi-step process involving various chemical signals; however, there is no evidence available to determine whether the elicitors are produced by the fungus and/or by the infected insect host [42]. In conclusion, an exciting and emerging issue to be addressed is to comprehend the crosstalk between fungal secondary metabolites, and/or other small signals, and the insect immune system, an interplay which is still waiting to be described in detail.

## 5. Immune Response of Insect Hosts

Insects display complex and sophisticated innate immunity, including both cellular and humoral responses, protecting themselves from pathogens [49,50]. The cellular response is largely mediated by hemocytes, which play a key role in the phagocytosis, encapsulation, and nodulation of pathogens [51,52]. The humoral response includes the recognition of pathogen-associated molecular patterns (PAMPs) on the surfaces of pathogenic microorganisms, which culminates with the induction of lectins; the prophenoloxidase cascade; and the biosynthesis of antimicrobial peptides (AMPs), a varied group of molecules [53,54,55]. Among the AMPs, several molecules with different structures have been reported to be activated by fungal pathogens, e.g., defensin [56], moricin [57], gallerimycin [58], and cecropin [59]. As stated before, dimorphic transition is a key step in fungal virulence since hyphal bodies are weaker inductors of the immune system than conidia and mycelia, acting by shedding epitopes to escape hemocyte encapsulation (i.e., they are cells lacking PAMPs); thus, fungal pathogens take advantage in the first battle in the hemocoel. The hyphal bodies grow exponentially in this nutrient-rich environment until achieving a critical threshold density, outnumbering circulating hemocytes, and then revert synchronously to a tissue-invasive mycelial cell phenotype [42,60]. At this last stage, the immune system may recognize PAMPs on the surface of the fungus, but the high density achieved makes this late response unsuccessful. The reverse-genetic-based study conducted by Feng et al. [25] reported that Δ*BbOpS1* delayed hemocyte encapsulation by 12 h, thus oosporein was linked with the evasion of insect host immunity. No functional information in this regard is available for the secondary metabolites biosynthesized by NRPS systems, except for some indirect clues contributed by phylogenetic analysis of BBA_07589, which has the same domain architecture as a siderophore synthetase exhibiting immunomodulatory activity in *Aspergillus fumigatus* [61]; thus, it was speculated that BBA_07589 can play a role in the evasion of the host immune system in *B. bassiana* [29].

Transcriptomics approaches in *B. bassiana*-infected larvae have demonstrated the induction of several immunity-related genes, including those for AMPs in *G. mellonella* [62], hemocytes and fat bodies from both *Ectropis obliqua* [63] and *Helicoverpa armigera* [64], and in *Ostrinia furnacalis* [65]. Furthermore, in *P. xylostella*, ~15% of the host genome was found to be enriched with genes for various immune processes [34]. Detailed dual qPCR assays have been conducted in the *B. bassiana*–*T. infestans* interacting system. The expression of six defensin genes in this insect host is modulated by two variants of the *limpet* transcription factor, and the silencing of both variants renders *T. infestans* more susceptible to *B. bassiana* infection [56]. The expression of humoral-immunity-related genes (defensins, as well as lectins and prophenol oxidase) is modulated by the fungal dose; however, at all the doses tested, the expression of the immune genes peaked later than that of the fungal NRPS genes [36,37], i.e., at a point when the infection process seems to be irreversible. Thus, the immune responses of the insect host try to limit or override the fungal infection, but once the fungus reaches the hemocoel, the insect has very little chance of surviving.

## 6. Concluding Remarks and Future Directions

The study of genes encoding NRPSs, PKSs, or hybrid NRPS–PKS proteins within insects is a useful approach for exploring the role of fungal secondary metabolites during pathogenesis. The NRPS expression signatures seem to be specific for fungal isolates, as suggested by qPCR studies [29,36,37]. However, transcriptomics approaches have failed to detect the entire NRPS expression pattern in vivo [66], even those that have used the same fungal isolate and host [29,34]. Both techniques are useful and complimentary for the study of toxic secondary metabolites, which have a changing expression pattern and are highly influenced by either the environment or the host. Transcriptomics, like other ‘omics tools, is very useful for discovery-driven research. This powerful technique provides excellent high-throughput approaches for studying an entire interacting system, tracing the expression of novel genes, and for the alternative splicing of already-known genes [35]; nevertheless, it can fail to detect poorly expressed genes. On the other hand, qPCR is a very sensitive technique; it can be used for either relative or absolute expression, and it allows the efficient quantification of known genes, even those with low expression. Potential predesigned panels for targeted gene expression for NRPSs, PKSs, and/or hybrid NRPS–PKSs would be very useful in future studies for unequivocally assigning roles to these genes as virulence factors in individual insects, hemocoel, and/or specific host tissues.

## Figures and Tables

**Table 1:** Current state of knowledge of the expression of synthetase genes involved in the biosynthesis of secondary metabolites by *Beauveria bassiana* within insects.

Gene Detected (Protein encoded, Family’s Acronym ^1^)	Insect Host	Technique Used [Reference]
*BbbeaS* (beauvericin synthetase, NRPS)	*Triatoma infestans* (Hemiptera: Reduviidae)	qPCR [36,37]
*BbbeaS* (beauvericin synthetase, NRPS)	*Plutella xylostella* (Lepidoptera: Plutellidae)	qPCR [29]
*BbbslS* (bassianolide synthetase, NRPS)	*Triatoma infestans* (Hemiptera: Reduviidae)	qPCR [36,37]
*BbbslS* (bassianolide synthetase, NRPS)	*Plutella xylostella* (Lepidoptera: Plutellidae)	qPCR [29]
*BbtenS* (tenellin synthetase, NRPS)	*Triatoma infestans* (Hemiptera: Reduviidae)	qPCR [36,37]
*BbtenS* (tenellin synthetase, NRPS)	*Plutella xylostella* (Lepidoptera: Plutellidae)	qPCR [29]
*BbopS1* (oosporein synthetase, PKS)	*Galleria mellonella* (Lepidoptera: Pyralidae)	qPCR [26]
*BBA_08222* (putative beauverolide synthetase, NRPS)	*Plutella xylostella* (Lepidoptera: Plutellidae)	qPCR [29]
*BBA_07611* (not detailed, NRPS)	*Plutella xylostella* (Lepidoptera: Plutellidae)	qPCR [29]
*BBA_07611* (not detailed, NRPS)	*Galleria mellonella* (Lepidoptera: Pyralidae)	RNA-seq [35]

^1^ NRPS, nonribosomal peptide synthetase; PKS, polyketide synthetase.

## Data Availability

Not applicable.
